# A Genome-Wide RNA Interference Screen Identifies a Role for Wnt/β-Catenin Signaling during Rift Valley Fever Virus Infection

**DOI:** 10.1128/JVI.00543-16

**Published:** 2016-07-27

**Authors:** Brooke Harmon, Sara W. Bird, Benjamin R. Schudel, Anson V. Hatch, Amy Rasley, Oscar A. Negrete

**Affiliations:** a Biotechnology and Bioengineering Department, Sandia National Laboratories, Livermore, California, USA; b Biosciences and Biotechnology Division, Lawrence Livermore National Laboratory, Livermore, California, USA; Icahn School of Medicine at Mount Sinai

## Abstract

Rift Valley fever virus (RVFV) is an arbovirus within the Bunyaviridae family capable of causing serious morbidity and mortality in humans and livestock. To identify host factors involved in bunyavirus replication, we employed genome-wide RNA interference (RNAi) screening and identified 381 genes whose knockdown reduced infection. The Wnt pathway was the most represented pathway when gene hits were functionally clustered. With further investigation, we found that RVFV infection activated Wnt signaling, was enhanced when Wnt signaling was preactivated, was reduced with knockdown of β-catenin, and was blocked using Wnt signaling inhibitors. Similar results were found using distantly related bunyaviruses La Crosse virus and California encephalitis virus, suggesting a conserved role for Wnt signaling in bunyaviral infection. We propose a model where bunyaviruses activate Wnt-responsive genes to regulate optimal cell cycle conditions needed to promote efficient viral replication. The findings in this study should aid in the design of efficacious host-directed antiviral therapeutics.

**IMPORTANCE** RVFV is a mosquito-borne bunyavirus that is endemic to Africa but has demonstrated a capacity for emergence in new territories (e.g., the Arabian Peninsula). As a zoonotic pathogen that primarily affects livestock, RVFV can also cause lethal hemorrhagic fever and encephalitis in humans. Currently, there are no treatments or fully licensed vaccines for this virus. Using high-throughput RNAi screening, we identified canonical Wnt signaling as an important host pathway regulating RVFV infection. The beneficial role of Wnt signaling was observed for RVFV, along with other disparate bunyaviruses, indicating a conserved bunyaviral replication mechanism involving Wnt signaling. These studies supplement our knowledge of the fundamental mechanisms of bunyavirus infection and provide new avenues for countermeasure development against pathogenic bunyaviruses.

## INTRODUCTION

Bunyaviruses constitute a diverse group of predominantly arthropod-vectored viruses of medical and agricultural importance with global distribution (1). Rift Valley fever virus (RVFV) is a particularly important bunyavirus due to its capacity to cause severe disease in humans, including retinal vasculitis, encephalitis, and fatal hepatitis with hemorrhagic fever, as well as lethal disease in economically valuable livestock species (2). Although currently endemic to the African continent and the Arabian Peninsula, there is a growing concern for the spread of RVFV into geographic locations outside regions where it is endemic (3). RVFV is also an agent of biodefense and agro-terrorism concern with the potential to cause social disruption requiring public health preparedness. For this reason, RVFV is classified as a category A priority pathogen by the National Institute of Allergy and Infectious Diseases, a high-consequence pathogen by the World Organization for Animal Health, and the third most dangerous animal threat by the United States Department of Agriculture Animal and Plant Health Inspection Service after avian influenza and foot-and-mouth disease. Currently, there are no FDA-approved therapies in the United States for use against RVFV or other pathogenic bunyaviruses. Lack of countermeasures against pathogenic bunyaviruses is partially due to gaps in knowledge of fundamental infection mechanisms and interactions between bunyaviruses and their host cells.

RVFV is an arthropod-borne virus that belongs to the Phlebovirus genus of the Bunyaviridae family. It is a spherical enveloped virus with three single-stranded RNA segments (L, M, and S segments) of negative or ambisense polarity that are encapsidated by the viral nucleocapsid (N) to form the ribonucleocapsid (RNP). The L segment encodes the viral RNA-dependent RNA polymerase (RdRp), which is packaged with the viral RNA genome in the virus particle. The M segment encodes two structural glycoproteins, Gn and Gc, and two nonstructural proteins, NSm1 and NSm2. The S segment is ambisense; it encodes the structural nucleoprotein N in the antisense orientation and encodes the nonstructural protein NSs in the sense orientation. RVFV NSs plays an important role in RVFV pathogenesis as it interferes with the cellular antiviral immune response by inhibiting host transcription, including synthesis of alpha/beta interferon mRNAs, and promoting degradation of the double-stranded RNA-dependent protein kinase (PKR) and TFIIH p62 (5–10).

The RVFV virions bind to cells and enter via pH-dependent caveola-mediated endocytosis (11). After viral uncoating, the viral RNP is released into the cytoplasm, where primary transcription occurs. Primary transcription of bunyaviral mRNA is primed by host-derived mRNA methylated cap structures that are obtained by a cap-snatching mechanism similar to that used by influenza A virus. Bunyaviral cap snatching involves two viral proteins: the N protein, which recognizes the 5′ cap and 10 to 18 nucleotides of cellular mRNAs, and the RdRp, which cleaves this fragment of mRNA and uses it to prime viral mRNA synthesis (12, 13). The 5′ cap on viral mRNA not only primes viral transcription but also protects the viral mRNA from host-mediated degradation and recruits host ribosomes for translation. Subversion of the host cell translation machinery and subsequent translation of these viral transcripts provide the protein products necessary for viral replication of the genome and further mRNA synthesis (secondary transcription).

Because viruses are obligate intracellular pathogens that rely on host cell machinery and pathways to complete their infection cycles, key cell signaling pathways regulating proliferation and differentiation responses are often prime targets of virus interaction and manipulation. A greater understanding of these interactions and how they relate to viral replication is necessary for development of effective targeted antiviral therapeutics. Genome-wide RNA interference (RNAi) screening is a powerful tool for functional genomics with the capacity to systematically perturb cellular pathways and comprehensively analyze host-pathogen interactions. Genome-wide RNAi screening has uncovered several previously uncharacterized cellular host factors involved in the infection of human immunodeficiency virus (HIV), West Nile virus, and influenza A virus (14–16). More recently, RNAi screening against the bunyaviruses Uukuniemi virus and RVFV has revealed roles for host proteins VAMP3 and the decapping enzyme Dcp2, respectively, during bunyavirus infection (12, 17). However, a large-scale RNAi screen against RVFV in human cells has yet to be described. To identify cellular factors required for RVFV infection in humans, we completed a genome-wide small interfering RNA (siRNA)-based screen, silencing ∼22,909 human genes in HeLa cells, and identified 381 genes whose knockdown reduced RVFV infection. After these 381 gene hits were grouped into functional clusters along cellular pathways, the Wnt signaling pathway was the most represented.

The Wnt/β-catenin pathway, or canonical Wnt pathway, is an evolutionarily conserved signaling cascade that involves activation of the transcriptional coactivator β-catenin (18). The Wnt/β-catenin signaling pathway is implicated in major physiologic cellular functions, such as proliferation, differentiation, and maintenance of pluripotency, while perturbations in this signaling cascade are associated with multiple types of cancer (19). The canonical pathway is best described in the OFF and ON state. In the OFF state, or in the absence of extracellular Wnt ligands, cytoplasmic β-catenin is sequentially phosphorylated by the β-catenin destruction complex (DC) composed of casein kinase 1a (CK1), glycogen synthase kinase 3 (GSK-3), the scaffold protein axin, and the tumor suppressor adenomatous polyposis coli (APC). Phosphorylated β-catenin is then targeted for ubiquitination and proteasomal degradation. In the ON state, Wnt ligands bind to the Frizzed (FZD) receptor, resulting in recruitment of the coreceptor low-density lipoprotein receptor-related protein 5 or 6 (LRP5 or -6, respectively), which is phosphorylated on its cytoplasmic tail, promoting the binding and polymerization of Disheveled protein (Dvl) and sequestration of axin. This inactivates the DC, resulting in accumulation of β-catenin in the cytoplasm and translocation to the nucleus. In the nucleus, β-catenin promotes transcription of genes related to proliferation and survival by acting as a coactivator for the T cell factor/lymphoid enhancer factor (TCF/LEF) family of transcription factors (18–22). Due to the importance of the Wnt/β-catenin pathway in diseases such as cancer, several preclinical therapeutic agents specifically targeting the Wnt pathway have been described, and some have recently entered clinical trials (20, 23).

The Wnt pathway has been a reported target of a variety of viruses, including human cytomegalovirus (HCMV) (24), hepatitis B virus (HBV) (25, 26), hepatitis C virus (HCV) (27), HIV (28), Epstein-Barr virus (EBV) and Kaposi's sarcoma–associated herpesvirus (KSHV) (29). HBV and HCV proteins activate Wnt signaling, and overactivation of Wnt signaling may contribute to hepatocellular carcinogenesis in chronic HBV/HCV infections (30, 31). The findings that pathogenic viruses manipulate the Wnt pathway for productive infection, together with the progress in the development of Wnt inhibitors for cancer treatments, suggest a new avenue for targeting these viruses by designing therapeutics that are host directed. This approach is illustrated in a recent study which showed that Wnt inhibitors can effectively block HCMV replication (32).

In this study, we validated the role of canonical Wnt signaling in RVFV infection using a number of different assays. We demonstrate activation of Wnt signaling by RVFV infection, enhancement of RVFV infection in cells prestimulated with Wnt ligands, and inhibition of RVFV infection using perturbations of Wnt signaling components at or downstream of the DC. We show similar results using wild-type RVFV and the distantly related bunyaviruses La Crosse virus (LCV) and California encephalitis virus (CEV), which indicates a conserved bunyaviral replication mechanism involving Wnt signaling. In the context of current literature, we postulate that bunyaviruses activate Wnt-responsive genes to regulate optimal cell cycle conditions for efficient viral replication (12, 13). We anticipate that this new understanding of the fundamental mechanisms of bunyavirus infection will aid in the design of efficacious broad-spectrum host-directed antiviral therapeutics.

## MATERIALS AND METHODS

### Cells, viruses, and reagents.

All cell lines were maintained in culture medium supplemented with 10% fetal bovine serum (FBS), 100 μg/ml penicillin, and 100 U/ml streptomycin (Life Technologies) at 37°C under 5% CO_2_. HeLa (human cervix carcinoma), 293T (human embryonic kidney), and A549 (human lung epithelial) cells were cultured in Dulbecco's modified Eagle's medium; Vero (African green monkey kidney) cells were cultured in minimum essential medium alpha. Primary human hepatocytes were obtained from ScienCell Research Laboratories and cultured using hepatocyte medium according to the company's instructions.

Wild-type Rift Valley fever virus strain ZH-501; NR-37378 (wild-type RVFV); recombinant vaccinia virus expressing green fluorescent protein (GFP), derived from the Western Reserve strain NR-624 (VacV) (33); La Crosse virus NR-540 (LCV); and California encephalitis virus strains BFS-283 and NR-89 (CEV) were obtained through the NIH Biodefense and Emerging Infections Research Resources Repository, NIAID, NIH. The recombinant RVFV vaccine strain MP12 generated to carry a green fluorescent protein (GFP) gene (RVFV MP12-GFP) in place of the NSs gene has been described previously (34). Authentic nonrecombinant RVFV strain MP12 was obtained from C. J. Peters (University of Texas Medical Branch). The recombinant vesicular stomatitis virus expressing GFP (VSV), derived from the Indiana serotype 1 strain (35), was a gift from Adolfo Garcia-Sastre (Mount Sinai School of Medicine). Studies using RVFV ZH-501 were conducted under biosafety level 3 containment using established standard operating procedures. Wild-type RVFV, RVFV MP12, RVFV MP12-GFP, LCV, CEV and VSV-GFP were propagated in Vero cells, while VacV-GFP was grown in BSC-40 cells. The viral titers for wild-type RVFV, RVFV MP12, RVFV MP12-GFP, VSV-GFP, CEV, and LCV were quantified in Vero cells by using a standard plaque assay consisting of an agarose overlay with crystal violet staining. VacV-GFP PFU were obtained by dilution of the virus and infection of BSC-40 cells without an agarose overlay (11). For real-time quantitative reverse transcription-PCR (qRT-PCR), RVFV MP12 and RVFV MP12-GFP stocks were first purified over a 20% sucrose cushion through ultracentrifugation.

Recombinant DKK-1, WIF-1, and purified Wnt3A (R&D Systems) were reconstituted in phosphate-buffered saline (PBS) containing 0.1% bovine serum albumin (BSA). All other inhibitors were from EMD Millipore and were initially resuspended in dimethyl sulfoxide (DMSO) followed by dilution in complete medium to obtain the final concentrations indicated. As a control for the DMSO-based inhibitors, cells were incubated with 50 μM DMSO alone, representing the highest concentration of DMSO used. Unless otherwise indicated, all experimental conditions were performed in triplicate, three or more times.

### High-throughput siRNA screening.

The primary screen was performed using the Qiagen human whole-genome set, the Agilent V11 Bravo automated liquid handler (Agilent Technologies), and the Multidrop Combi (Thermo Scientific). Pooled siRNAs (4 per target, 50 nM final concentration) were complexed with Lipofectamine RNAiMAX (catalog number 13778500; 0.1 μl/well in 0.5 μl Opti-MEM; Life Technologies). After 20 min, HeLa cells (3,000 cells/well) were added to each well. Cells were infected with RVFV MP12-GFP at a multiplicity of infection (MOI) of 1 at 48 h posttransfection (hpt) for 3 h; then, the cells were washed with PBS and were incubated in complete medium overnight. At 24 h postinfection (hpi), alamarBlue (AB) and GFP fluorescence were measured to calculate normalized infection values as previously described (11). In all of our assays, we used a fluorescence-based readout of AB which has been demonstrated to be highly sensitive (as few as 50 cells in a 96-well plate can be detected with relatively short incubations) and linear with a signal that is proportional to cell number. To verify manufacturer claims, cell titration experiments with the AB reagent were performed during screen optimization and a linear increase in signal with increasing concentrations of seeded cells in 384- and 96-well plates was demonstrated. For every plate in the initial screen, secondary screen, and follow-up analysis, AB cell viability tests were performed. Any siRNA-containing wells that demonstrated toxicity (1.25× reduction in 560 nm excitation and 590 nm emission(Ex:560nm/Em:590nm) fluorescence compared to wells without siRNA) were removed/eliminated from the hit list and not included in the presented analysis. Sample means and standard deviations (SDs) of negative controls were calculated plate-wise and used to set a Z-score threshold value of −3. To be considered a hit, each replicate needed to be 3 SDs from the mean.

### Secondary screen and final hit selection.

To test the hits identified from the primary screen, the siRNA hits from the master plates were picked and spotted on 3 new daughter plates using the Beckman-Coulter BioMek NX with Span-8, and the screen was performed at Sandia National Laboratories using the Bio-Tek EL406 microplate washer and dispenser using the reverse transfection, infection, and analysis assays described above.

### Final hit selection data analysis.

Final hit selection was based on a uniformly minimal variance unbiased estimate (UMVUE) of strictly standardized mean differences (SSMD) using the paired method formula,$\frac{\Gamma (\frac{n\;-\;1}{2})}{\Gamma (\frac{n\;-\;2}{2})}\sqrt{\frac{2}{n\;-\;1}\;}\frac{\overset{¯}{d_{i}}}{s_{i}}$, where *n* is the number of replicates, *d_i_* is the sample mean, and *s_i_* is the SD for the *i*th siRNA. A meaningful and interpretable SSMD-based criterion for classifying the size of siRNA effects is as follows: |SSMD| ≥ 5 for extremely strong, 5 > |SSMD| ≥ 3 for very strong, 3 > |SSMD| ≥ 2 for strong, 2 > |SSMD| ≥ 1.645 for fairly strong, 1.645 > |SSMD| ≥ 1.28 for moderate, 1.28 > |SSMD| ≥ 1 for fairly moderate, 1 > |SSMD| ≥ 0.75 for fairly weak, 0.75 > |SSMD| ≥ 0.5 for weak, 0.5 > |SSMD| ≥ 0.25 for very weak, and |SSMD| > 0.25 for extremely weak effects (36). Therefore, a threshold of −1.30 was set as a cutoff value to identify moderate to extremely strong siRNA hits that reduced RVFV MP12-GFP infection.

### β-Catenin siRNA treatment.

Silencer select siRNAs (catalog number AM16708) targeting the β-catenin gene (Life Technologies) and scrambled control siRNA (AllStars negative-control siRNA; Qiagen) were transfected into HeLa or 293T cells using Lipofectamine RNAiMAX. At 60 hpt, cells were infected with indicated viruses (MOI of 1) for 3 h, followed by a PBS wash and incubation in complete medium overnight. AB and GFP fluorescence signals were then measured to calculate normalized infection values.

### Western blot analysis.

Samples were lysed and analyzed by Western blotting as previously described (11). Primary antibody for β-catenin knockdown experiments was rabbit polyclonal anti-β-catenin (H-102; Santa Cruz Biotechnology). Primary antibody to measure levels of β-catenin activation was non-phospho (active) β-catenin (Ser33/37/Thr41) (D13A1) rabbit monoclonal antibody (Cell Signaling). All blots were reprobed with rabbit antiactin polyclonal antibody (Novus). The relative reduction index (RI) was calculated as the quotient of the densitometry signal for the target protein band divided by that for actin, which was then normalized by the ratio obtained with scrambled siRNA or no-ligand controls (considered to be 1).

### β-Catenin transcriptional reporter assay.

TOPflash (TF; TCF/LEF-1 reporter plasmid) or FOPFlash (contains mutated TCF/LEF-1 binding sites) was cotransfected with pcDNA3.1-GFP or pcDNA3.1-mKate (used with GFP-expressing viruses; Thermo Fisher Scientific) to serve as an internal transfection control and control for effects on overall gene expression, using TransIT-LT1 transfection reagent (Mirus Bio) according to the manufacturer's protocol. Luciferase activity was assayed using the Bright-Glo luciferase reporter assay kit (Promega, Madison, WI) according to the manufacturer's protocol. Intensity is shown as relative light units (RLU). Cells were treated with Wnt3A or indicated virus 18 hpt. For cells cotransfected with TOPflash/pcDNA3.1-GFP/mKate and treated with 100 ng/ml Wnt3A or indicated virus concentrations, the luciferase activity measured was normalized to the GFP (Ex:488nm/Em:510nm) or mKate (Ex:588nm/Em:633nm) expression signal. Fold activation was calculated by dividing the relative luciferase activity of treated/infected cells by that of untreated cells. The relative percentage of luciferase activity was determined by subtracting the uninfected/unstimulated cells as background and taking the no-inhibitor (NI)/DMSO condition and infected/Wnt3A-stimulated samples as 100%. For TOPflash-expressing 293T cells treated with inhibitors, all conditions included treatment of cells with each inhibitor concentration as indicated, prior to and during treatment with Wnt3A, with no virus (mock infection), and with indicated viruses. Inhibitors had no effect on luciferase activity in the mock-infected wells, and the luciferase activity from these wells was subtracted as background.

### Quantitative real-time RT-PCR.

Total RNA was isolated and purified with the ZR RNA MiniPrep extraction kit (Zymo Research), and cDNA was made using the SuperScript VILO cDNA synthesis kit (Life Technologies). The primer and probe sets and TaqMan Gene Expression master mix were purchased from Applied Biosystems, and gene expression measurements were analyzed on a Bio-Rad CFX96 real-time PCR machine. Each experimental condition was run in triplicate, and the relative amount of target gene mRNA was normalized to glyceraldehyde-3-phosphate dehydrogenase (GAPDH) mRNA.

### Infection assays in Wnt ligand-stimulated cells.

Cells were incubated in medium alone or at increasing concentrations of Wnt3A for 20 h and then treated for 3 h with virus. Cells were then washed with PBS and incubated in complete medium at 37°C for 14 to 16 h. AB and GFP fluorescence signals were then measured to calculate normalized infection values. Levels of virus infection enhancement were measured by fold changes between Wnt-stimulated/infected conditions and unstimulated/infected controls. These experiments were performed in triplicate for each cell type 3 or more times. For pretreatment of HeLa cells with Wnt3A prior to infection with wild-type RVFV, HeLa cells were untreated or treated with 50 ng/ml of Wnt3A for 20 h prior to and during 36 h of mock infection or infection with wild-type RVFV (MOI of 0.1). After 36 h, supernatants were collected and virus titers were quantified by a standard plaque assay on Vero cells.

### Inhibitor treatment, time-of-addition experiments, plaque assays, and flow cytometry.

For most experiments, cells were incubated with medium alone (no inhibitor [NI]), DMSO, or individual inhibitors for 1 h prior to and during incubation with viruses or Wnt3A. If infection continued overnight, virus was removed, cells were washed with PBS, and inhibitors were added back in complete medium for 16 h before relative percent infection was measured. For time-of-addition experiments, pretreatment samples were incubated with indicated concentrations of inhibitors for 1 h prior to and during 3 h of infection. Virus and inhibitors were then removed by washing once with PBS, and cells were incubated in compete medium alone overnight (no inhibitor added back). Alternatively, untreated cells were incubated with virus for 1 h, washed with PBS to remove unbound virus, and then incubated with inhibitors in complete medium for 16 h before relative percent infection was measured. To measure viral titers of wild-type RVFV, RVFV MP12, CEV, and LCV, supernatants were removed at the indicated times postinfection and titers were determined by a standard plaque assay on Vero cells. For flow cytometry experiments, 293T cells were incubated with indicated concentrations of inhibitors for 1 h prior to and during the 16-h infection with virus (MOI of 3). Viral antigens were detected by immunofluorescence as previously described (11). For RVFV MP12-infected samples, anti-RVFV mouse polyclonal antibody (provided by Robert Tesh [UTMB]) was used as the primary antibody and Alexa Fluor 488 goat anti-mouse IgG (Invitrogen) was used as a secondary antibody. Cells were analyzed using an Accuri C6 flow cytometer with FCS Express software (De Novo Software). Cells were counted as infected if their FL-1 fluorescence was greater than that of the untreated or DMSO-treated, uninfected cells (see Fig. 4G, red histograms). The quantity of cells infected is given as percent cells infected. The average (± standard deviation) from four independent experiments is shown. Uninfected cells were similarly probed with primary and secondary antibody to control for any nonspecific binding.

### Statistical analysis.

Raw data for infection assays measured by GFP fluorescence were compared using a two-tailed *t* test for each individual experiment. Values obtained with inhibitors suspended in DMSO were compared to DMSO-treated samples, and values obtained with inhibitors suspended in water were compared to infected, untreated samples. For siRNA-treated samples, values obtained from wells transfected with indicated siRNA constructs were compared to values obtained with wells transfected with scrambled siRNA on the same plate. For plate assays, untreated and DMSO-treated controls, or scrambled siRNA and GFP siRNA, were included on every plate, with no virus, RVFV MP12-GFP, VacV, and VSV. Inhibitor-treated or siRNA-transfected cells that were infected with wild-type RVFV, RVFV MP12, or RVFV MP12-GFP were compared to control samples infected with the same virus, as was the case for VacV and VSV. For qRT-PCR, the threshold cycle (*C_T_*) values obtained with virus-infected samples were compared to *C_T_* values of mock-infected samples using a two-tailed *t* test for each individual experiment. *P* values were considered significant when they were <0.05 (*) and very significant when they were <0.01 (**), <0.001 (***), or <0.0001 (****). The *P* values shown in the figures and text were based on the highest *P* values obtained from three independent experiments.

## RESULTS

### Genome-wide RNAi screening reveals a role for Wnt/β-catenin signaling in RVFV infection.

**FIG 1:**
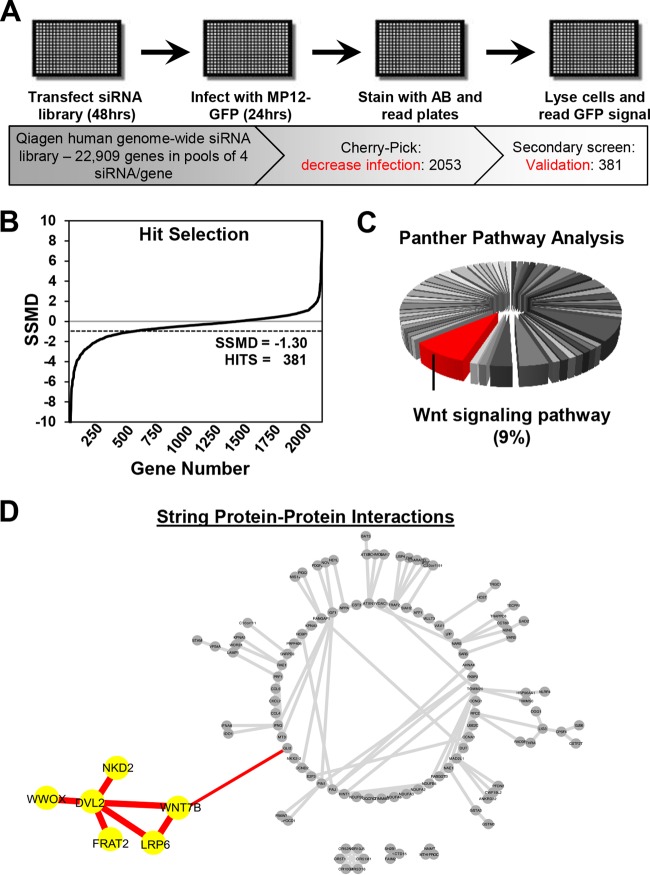
Genome-wide RNAi screening identifies a role for Wnt/β-catenin signaling in RVFV infection. (A) Schematic of the RNAi screen. (B) Hit selection was based on strictly standardized mean difference (SSMD) statistics. The SSMD threshold was set to −1.30, which accounted for 381 gene hits that reduced infection upon their knockdown. (C) The 381-gene hit list was analyzed through a bioinformatics online resource termed Panther, and 89 of those genes were clustered into one of 56 cellular pathways represented by pie sections in the chart. Nine percent of the 89 genes clustered to the Wnt signaling pathway (red). (D) The RNAi screening hit list was also subjected to STRING bioinformatics, a search tool for retrieval of interacting genes/proteins from a database of known and predicted protein interactions and networks. Cytoscape data visualization software was used to display STRING results. Proteins without interactions and the ubiquitin C node were removed and not shown in the radial graph. Wnt signaling genes/proteins and interactions identified by these bioinformatics tools are highlighted in red or in red and yellow.

To identify novel cellular factors involved in RVFV infection, we conducted a comprehensive high-throughput screen using genome-wide RNAi. The screen was performed in HeLa cells by targeting human genes with pools of four different siRNAs against each gene. To easily characterize the percentage of cells infected in a population, we used the recombinant GFP reporter virus of the attenuated RVFV strain MP12 (RVFV MP12-GFP) for the screen (11, 34). The GFP gene in the RVFV MP12-GFP reporter virus replaced the nonstructural protein NSs, and GFP expression serves as a sensitive readout of viral infection (34, 37). The RNAi screen was carried out as illustrated in Fig. 1A and detailed in Materials and Methods. We designed a screening method in which we seeded HeLa cells into 384-well plates, reverse transfected the cells with siRNA in triplicate for 2 days, and then infected the cells with RVFV MP12-GFP at an MOI of 1. One day later, the cells were analyzed with a quantitative cell viability reagent (alamarBlue [AB]) and then were lysed and assayed for GFP expression as a marker of virus replication (37). GFP signals were normalized to cell number values, and control experiments were set to 100% infection. For this primary screen, hit selection was based on Z-score statistics; this method was used because the large number of siRNAs typically produces a frequency distribution with an approximate Gaussian curve, albeit with a slight skew (38). Using a Z-score threshold set at −3, or 3 standard deviations (SDs) from the mean, we restricted our genes of interest to only those that decreased RVFV MP12-GFP infection and identified 2,053 gene targets.

To validate the primary hits, the 2,053 siRNAs were replated and rescreened using the same transfection and infection protocol described above. Hit selection for the secondary screen was based on strictly standardized mean differences (SSMD), which minimizes the rates of false discovery and false nondiscovery in siRNA-based screens and is calculated based on controls and replicates rather than a large sample size needed for Z-score statistics (36). A SSMD score was calculated for each gene in the secondary screen using the means of the replicates after prior normalization using plate means. The threshold was set to −1.30, which resulted in 381 gene hits that reduced infection upon their knockdown (Fig. 1B). The final hit list is shown in Table S1 in the supplemental material.

To extract information regarding critical processes in infection, we performed bioinformatics analysis on the hit list. Final hits were functionally clustered using the Panther pathway classification system (39). Using this method, 89 of the total 381 genes were grouped into 56 separate pathways (see Table S1 in the supplemental material, Panther worksheet), and the Wnt signaling pathway was the most represented pathway with 9% (8 of 89) of the genes (Fig. 1C). A second bioinformatics analysis was performed using DAVID, a public database that assigns hit lists to ‘functional annotation clusters’ or sets of proteins that share common annotations (40). The top 20 annotation clusters are shown in Table S1 in the supplemental material (DAVID worksheet), and again, the Wnt pathway was among the most represented pathways in the hit list with the second best enrichment score. Last, the hit list was subjected to the protein-protein interaction database STRING, to identify proteins known or predicted to interact within the list (41). As shown in Fig. 1D, among the most represented cluster of interacting proteins are those related to the Wnt pathway.

**FIG 2:**
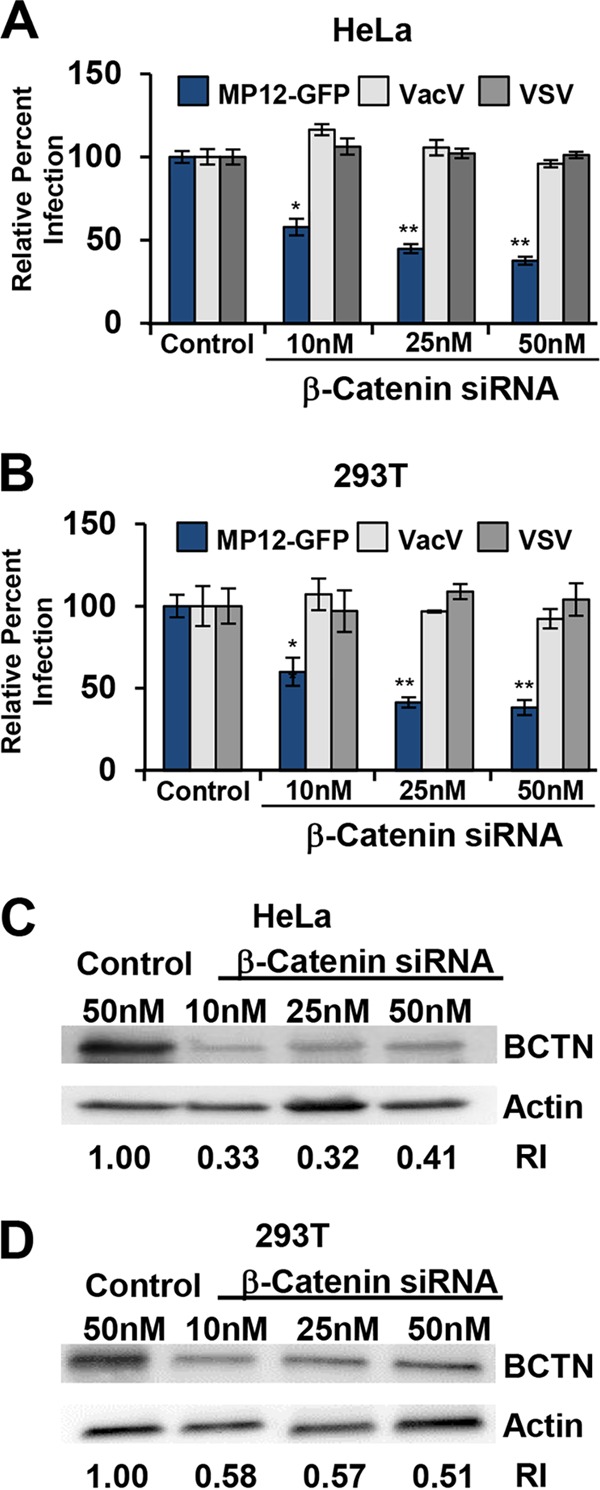
Canonical Wnt signaling plays a significant role in RVFV infection. (A and B) HeLa cells (A) or 293T cells (B) were transfected with siRNAs targeting β-catenin or 50 nM control siRNA and then infected with GFP reporter viruses (MOI of 1). The percentage of infection was determined by taking control and infected samples as 100%. Shown are the means (±SDs) for 3 independent experiments performed in triplicate (**, *P* < 0.01; *, *P* < 0.05). (C and D) Transfected HeLa cells (C) or 293T cells (D) were washed and lysed at 60 hpt, and whole-cell lysates were analyzed by Western blotting. The relative reduction index (RI) was measured as described in Materials and Methods. The data represent one of 3 experiments with similar results.

To verify the role of Wnt signaling in RVFV infection and distinguish between canonical Wnt signaling involving β-catenin and β-catenin-independent noncanonical Wnt pathways, we transfected HeLa cells with increasing concentrations of siRNA targeting β-catenin, and these cells were mock infected or infected with RVFV MP12-GFP, vaccinia virus expressing GFP (VacV), or vesicular stomatitis virus expressing GFP (VSV) at an MOI of 1. Infection with RVFV MP12-GFP was reduced in a dose-dependent manner in siRNA-transfected HeLa cells (Fig. 2A) and 293T cells (Fig. 2B). The decrease in RVFV MP12-GFP infection correlated with the decreased steady-state levels of β-catenin at 60 h posttransfection (hpt), as detected by immunoblotting (Fig. 2C and D). Although β-catenin was not identified as a hit in our screen, we found that the best knockdown of β-catenin was observed at 60 h, and there was very little knockdown at 48 h (when cells were infected for the screen). Accordingly, RVFV MP12-GFP infection was reduced at 60 hpt with β-catenin siRNA (Fig. 2C and D) and not at 48 hpt (data not shown). Infection of HeLa cells (Fig. 2A) or 293T cells (Fig. 2B) with VacV or VSV was unaffected by downregulation of β-catenin. Interestingly, each of these viruses replicates in the cell cytoplasm, and yet only RVFV MP12-GFP was inhibited by a reduction in the canonical Wnt signaling protein β-catenin, suggesting a specific role for Wnt/β-catenin signaling in RVFV infection.

### RVFV induces Wnt/β-catenin signaling, and preactivation of Wnt signaling enhances RVFV infection.

**FIG 3:**
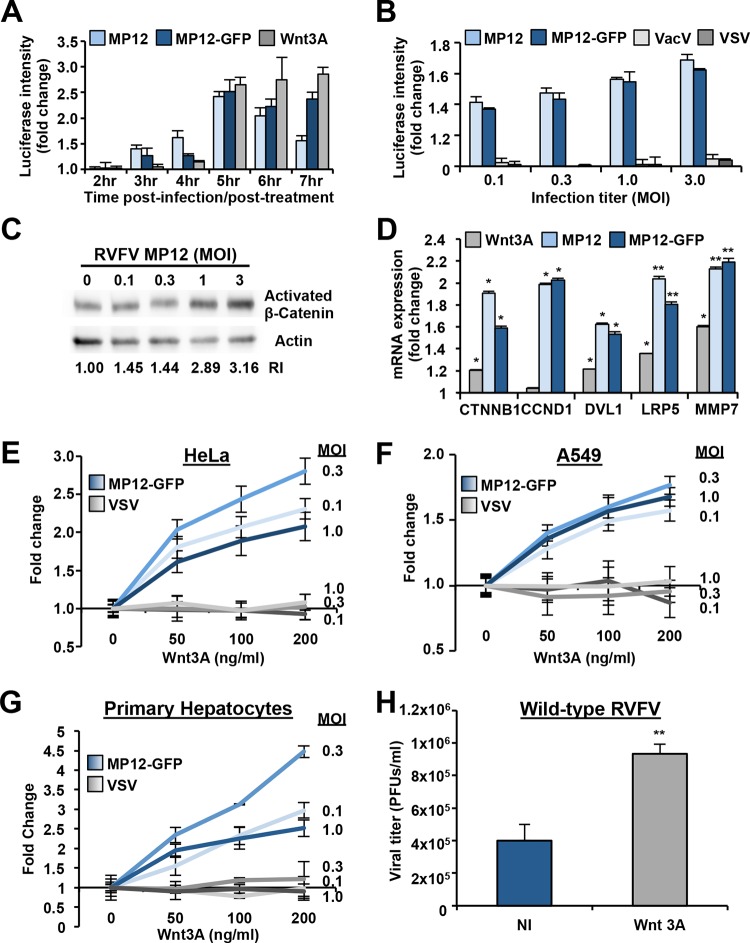
RVFV infection induces Wnt/β-catenin signaling, and preactivation of the canonical Wnt pathway enhances RVFV replication. (A and B) 293T cells were transfected with the TCF/LEF-1 Luc reporter construct TF and pcDNA3.1-GFP or pcDNA3.1-mKate reporter plasmids. (A) At 18 hpt, cells were treated with positive-control Wnt3A (100 ng/ml) or virus (MOI of 3) for the indicated time periods. (B) At 18 hpt, cells were infected with virus at the indicated MOIs (all viruses were used at the same MOIs as determined by plaque assay). At 5 hpi, luciferase activity was measured and normalized to expression of GFP (RVFV MP12) or mKate (RVFV MP12-GFP, VacV, and VSV), and fold activation was calculated by dividing the relative luciferase activity of treated/infected cells by that of untreated cells. Data are presented as means ± SDs. (C) 293T cells were infected with RVFV at indicated MOIs, and whole-cell lysates were analyzed by Western blotting at 4.5 hpi. The top membrane was probed with activated β-catenin antibody, and the bottom membrane was probed with anti-actin antibody. Representative results are shown (*n* = 3). (D) Gene expression analysis was performed using quantitative RT-PCR for the β-catenin (*CTNNB1*), cyclin D1 (*CCND1*), *DVL1*, *LRP5*, or matrix metalloproteinase-7 (*MMP7*) gene after 20 h of treatment with 50 ng/ml Wnt3A or 4.5 h of infection with virus (MOI of 1) in A549 cells. Each experimental condition was run in triplicate, and the fold change was determined by dividing the average of the infected samples by the average of uninfected samples. (E to G) HeLa cells (E), A549 cells (F), or primary hepatocytes (G) were pretreated with indicated concentrations of Wnt3A for 20 h and then infected at an MOI of 0.1, 0.3, or 1 with RVFV MP12-GFP (blue-shaded lines) or VSV (gray-shaded lines) as described in Materials and Methods. Levels of virus infection enhancement are indicated by fold changes compared to untreated/infected controls. (H) HeLa cells were preincubated with NI or 50 ng/ml Wnt3A for 20 h followed by infection with wild-type (WT) RVFV at an MOI of 0.1. Wnt3A was also present during the infection. Supernatants were collected at 36 hpi, and viral titers were measured by plaque assay on Vero cells. Three independent experiments were performed in triplicate. Data are presented as means ± SDs (**, *P* < 0.01; *, *P* < 0.05).

To further investigate the role of Wnt/β-catenin signaling in RVFV infection, the TCF/LEF luciferase (Luc) reporter construct TOPflash (TF) was used to determine whether RVFV infection induces β-catenin-dependent transcriptional activity. 293T cells were transiently transfected with TF for 18 h and subsequently infected with the nonrecombinant RVFV strain MP12 (RVFV MP12) or RVFV MP12-GFP at an MOI of 1. As a positive control, cells were also separately treated with the canonical Wnt3A ligand. The cells were then analyzed for Luc expression between 2 and 7 h postinfection (hpi) or posttreatment. It has been previously demonstrated that RVFV proteins begin to accumulate at 2 hpi and steadily increase within this measurement time frame (8). As shown in Fig. 3A, RVFV MP12 and RVFV MP12-GFP induced β-catenin reporter activity to levels similar to those induced by Wnt3A stimulation at 5 hpi. The reporter activity remained elevated after 6 and 7 h of treatment with Wnt3A or infection with RVFV MP12-GFP, whereas β-catenin reporter activity peaked at 5 hpi and decreased thereafter in cells infected with RVFV MP12. Unlike RVFV MP12-GFP, RVFV MP12 contains the nonstructural protein NSs. Since NSs is known to downregulate cellular transcription, NSs is likely responsible for decreased β-catenin/TCF/LEF complex reporter activity in late stages of infection. However, activation is not dependent on NSs since RVFV MP12 and RVFV MP12-GFP (ΔNSs) infections similarly induced β-catenin-dependent transcriptional activity. RVFV MP12 and RVFV MP12-GFP infections were also shown to similarly promote β-catenin reporter activation across a range of infectious doses measured at 5 hpi (Fig. 3B). In contrast to RVFV MP12 and RVFV MP12-GFP, VSV and VacV did not activate Luc expression through the TCF/LEF promoter using infectious titers equivalent to those of RVFV MP12 and RVFV MP12-GFP (Fig. 3B). Therefore, RVFV seems to specifically induce Wnt signaling.

Another way to measure Wnt/β-catenin activation is through the analysis of β-catenin phosphorylation (4). Again, in the OFF state of canonical Wnt signaling, CK1 phosphorylates β-catenin specifically at Ser45. This phosphorylation event primes β-catenin for subsequent phosphorylation by GSK-3 at Ser33, Ser37, and Thr41, and phosphorylated β-catenin is then targeted for ubiquitination and proteasomal degradation. In the ON state, there is a rise in the stabilized (nonphosphorylated) form of β-catenin (21, 22). Therefore, using an antibody designed to specifically detect nonphosphorylated sites Ser33/37/Thr41 of β-catenin, we probed 293T cell lysates after infection with RVFV MP12 at a range of infectious doses for functionally active β-catenin (19, 21). As shown in Fig. 3C, RVFV MP12 infection of cells with an MOI of 1 or 3 for 4.5 h induced an ∼3-fold increase in levels of activated β-catenin.

Next, the expression of endogenous Wnt/β-catenin target genes was examined in RVFV MP12- or RVFV MP12-GFP-infected cells. As a control, 50 ng/ml Wnt3A was used to treat the cell cultures and induce expression of β-catenin-regulated genes. Quantitative real-time RT-PCR (qRT-PCR) analysis was used to measure the mRNA expression levels of β-catenin, cyclin D1, Dvl, LRP5, or matrix metalloproteinase-7 (MMP7) after infection with RVFV MP12 or RVFV MP12-GFP (MOI of 1) or treatment with Wnt3A in A549 cells. The GFP reporter signal from RVFV MP12-GFP was readily observed between 5 and 6 hpi in this cell type, ensuring that viral mRNA transcription and translation occurred within this measurement time frame (42, 43). RVFV MP12 and RVFV MP12-GFP infection led to significant upregulation of expression of all five genes compared to that in mock-infected cells (Fig. 3D).

To determine if Wnt signaling positively regulates RVFV infection, we treated HeLa cells (Fig. 3E) and A549 cells (Fig. 3F) with increasing concentrations of Wnt3A prior to infection. In both HeLa and A549 cells, RVFV MP12-GFP viral infection was significantly enhanced compared to that with VSV when various infectious doses were used. To verify these results in the context of primary cell infection, primary hepatocytes were similarly treated with Wnt3A prior to infection with RVFV MP12-GFP or VSV. Hepatocytes are a prime *in vivo* target of RVFV infection in several animal models and in the more severe consequences of human RVFV infection that leads to fatal hepatitis with hemorrhagic fever (44). We found that stimulation of primary hepatocytes with Wnt3A induced a greater-than-4.5-fold increase in RVFV MP12-GFP (MOI of 0.3) infection, while VSV infection remained unchanged with similar concentrations and infection conditions (Fig. 3G).

Finally, in order to determine whether or not preactivation of Wnt signaling would enhance infection with fully virulent wild-type RVFV, HeLa cells were pretreated with Wnt3A for 20 h prior to mock infection or infection with wild-type RVFV strain ZH-501 (MOI of 0.1), and the extent of infection was quantified by plaque assay of the supernatants collected from virus-infected cells. Wild-type RVFV infection was enhanced in Wnt3A-treated cells in comparison to untreated cells as demonstrated by a significant increase in the number of plaques (Fig. 3H). All together, these results demonstrate that RVFV infection induces Wnt/β-catenin transcriptional activity and preactivation of the Wnt pathway enhances RVFV infection. The results with wild-type RVFV and RVFV MP12 parallel those seen with RVFV MP12-GFP (ΔNSs), further strengthening the case for NSs-independent activation of the Wnt signaling pathway.

### Inhibitors of Wnt/β-catenin signaling downstream of membrane receptor complex block RVFV infection at a postentry step.

**FIG 4:**
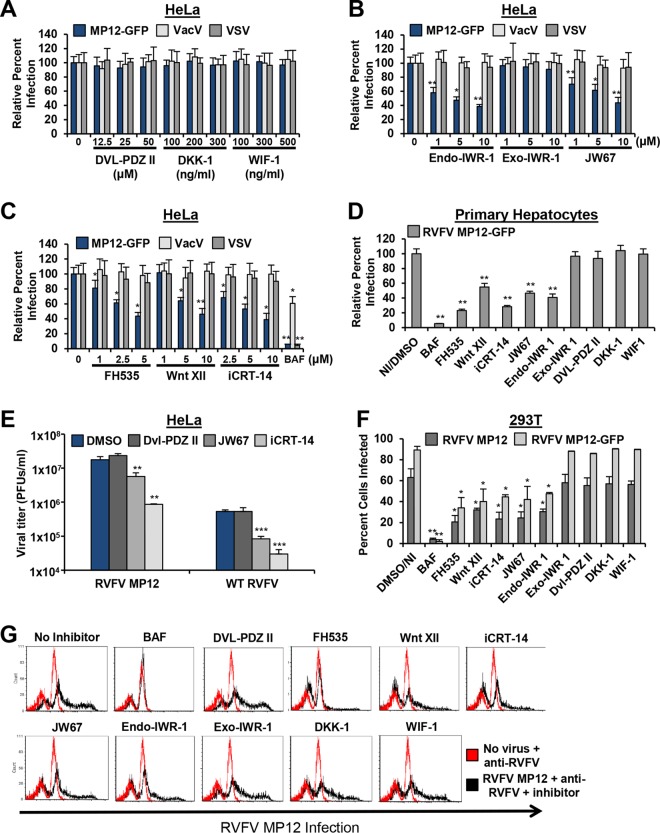
Inhibitors that block Wnt signaling downstream of the membrane receptor complex inhibit RVFV infection. (A to C) HeLa cells were pretreated with indicated inhibitors for 1 h. BAF (100 nM) was used as a control for inhibition of RVFV infection. The inhibitors were also present during 3 h of incubation with GFP reporter viruses (MOI of 1) and during overnight incubation. (D) Primary human hepatocytes were pretreated with no inhibitor/50 μM DMSO, 5 μM FH535, 50 μM Dvl-PDZ II, 300 ng/ml DKK-1, 500 ng/ml WIF, or 10 μM Wnt XII, iCRT-14, JW67, Endo-IWR-1, or Exo-IWR-1 for 1 h prior to and during 3 h of infection with RVFV-GFP (MOI of 1) and during overnight incubation. The percentage of infection was determined by taking untreated (NI) or DMSO-treated and infected samples as 100% infected (0 μM). Three independent experiments were performed in triplicate. Data are presented as means ± SDs (**, *P* < 0.01; *, *P* < 0.05). (E) HeLa cells were preincubated for 1 h with 50 μM DMSO (blue), Dvl-PDZ II (dark gray), JW67 (medium gray), or iCRT-14 (light gray) followed by infection with RVFV MP12 or WT RVFV at an MOI of 0.1. The inhibitors were also present during the infection. Supernatants were collected at 36 hpi, and viral titers were measured by plaque assay on Vero cells. Data are presented as means ± SDs (*n* = 3) (****, *P* < 0.0001; ***, *P* < 0.001; **, *P* < 0.01). (F and G) 293T cells were preincubated for 1 h with 100 nM BAF, 5 μM FH535, 10 μM Wnt XII, 10 μM iCRT-14, 10 μM JW67, 10 μM Endo-IWR, 10 μM Exo-IWR, 50 μM Dvl-PDZ, 300 μg/ml DKK-1, or 500 μg/ml WIF-1. The cells were then infected with RVFV MP12 or RVFV MP12-GFP at an MOI of 3 for 16 h in the presence of inhibitors. Infection was measured by flow cytometry using anti-RVFV polyclonal antibodies or GFP expression for RVFV MP12-GFP. Shown are the means (±standard deviations) from four independent experiments for each cell type (**, *P* < 0.01; *, *P* < 0.05). (G) Red histograms represent uninfected 293T cells that were left untreated or were treated with DMSO; black histograms represent RVFV MP12-infected 293T cells treated with the indicated inhibitors. The data shown are representative results from four similar experiments.

Wnt/β-catenin is a major signaling pathway with significant implications in a broad range of diseases, including degenerative diseases, metabolic diseases, and cancer. As such, the Wnt/β-catenin signaling pathway has been a prime target for pharmacological research and development. Multiple antagonists and small-molecule inhibitors that block at various points along the Wnt pathway have been identified, including those that target the membrane receptor complex, those that stabilize the DC, and those that interfere with activated β-catenin (20). The recombinant proteins DKK-1 and WIF-1 and the small-molecule inhibitor Dvl-PDZ domain inhibitor II (Dvl-PDZ II) block signaling from the Wnt receptor complex to the DC, and so the DC remains intact and β-catenin is degraded. DKK-1 functions as an antagonist of canonical Wnt signaling by binding to coreceptors LRP5 and -6, preventing interaction of LRP5 and -6 with Wnt-FZD complexes. WIF-1 is a secreted protein that binds to Wnt ligands and inhibits their activity, while Dvl-PDZ II is a compound that targets the PDZ domain of Dvl to disrupt the interaction of Dvl with the FZD receptor, preventing sequestration of axin (45). Shown in Fig. 4A, treatment of HeLa cells with increasing concentrations of DKK-1, WIF-1, and Dvl-PDZ II was unable to reduce infection of RVFV MP12-GFP, VacV, or VSV. Similar results were seen in A549 cells (data not shown).

To determine the role of the DC in RVFV infection, cells were treated with Endo-IWR-1, Exo-IWR-1, and JW67. The tankyrase inhibitors JW67 and Endo-IWR-1 prevent ADP-ribosylation-dependent axin degradation, resulting in stabilization of the DC and phosphorylation of β-catenin. Exo-IWR-1 is an inactive stereoisomer of Endo-IWR-1. Treatment of cells with these inhibitors reduced RVFV MP12-GFP infection in a dose-dependent manner, with a greater than 50% decrease in RVFV MP12-GFP infection (Fig. 4B). As expected, Exo-IWR-1 treatment did not inhibit RVFV MP12-GFP, and none of the inhibitors that stabilize the DC affected VacV or VSV infection, indicating the specificity of Endo-IWR-1 and JW67. Additionally, small-molecule inhibitors that interfere with the interaction of β-catenin with TCF/LEF transcription factors (FH535, Wnt pathway inhibitor XII [Wnt XII], and iCRT-14) reduced RVFV MP12-GFP infection but did not diminish VacV or VSV infections in HeLa cells (Fig. 4C) and A549 cells (data not shown). The same Wnt signaling inhibitors that blocked infection in cell lines also reduced RVFV MP12-GFP infection in *in vivo* relevant primary hepatocytes (Fig. 4D).

Using the fluorescence-based plate reader assay that measures GFP fluorescence and cell viability, we were able to control for the specificity and efficacy of the small-molecule inhibitors by including VacV and VSV infection to exclude nonspecific effects. To look more directly at the effect of the Wnt inhibitors on viral growth, we measured viral titers of both wild-type RVFV and RVFV MP12 in the presence of inhibitors. When HeLa cells were treated with the Wnt inhibitors iCRT-14 and JW67, there was a significant decrease in RVFV MP12 and wild-type RVFV viral titers as measured by plaque assay (Fig. 4E). However, no change in viral titer was observed when cells were treated with the Wnt receptor antagonist Dvl-PDZ II (dark gray bar), as was observed with RVFV MP12-GFP. Similar observations were made when flow cytometry was used to measure the percentage of inhibitor-treated cells infected with RVFV MP12 or RVFV MP12-GFP. Inhibitors that block Wnt signaling upstream of the DC had no effect on infection in 293T cells (Fig. 4F and G), while inhibitors that stabilize the DC or antagonize β-catenin interactions with transcription factors significantly reduced the percentage of 293T cells (Fig. 4F and G) infected with RVFV MP12 or RVFV MP12-GFP.

**FIG 5:**
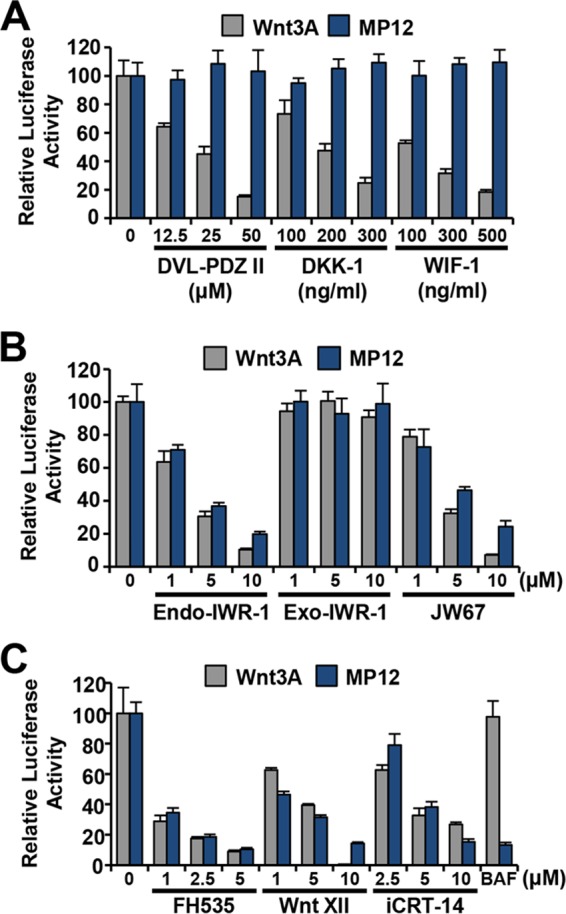
RVFV-induced β-catenin transcriptional activity is blocked by inhibitors that act downstream of the membrane receptor complex. 293T cells were transfected with TF and pcDNA3.1-GFP. At 18 hpt, cells were treated with the indicated inhibitors for 1 h. The inhibitors were also present during the 4.5 h of incubation with RVFV MP12 (MOI of 3) or 20 h of incubation with Wnt3A (100 ng/ml). Luciferase activity was measured and normalized to GFP expression, and relative luciferase activity was determined by taking untreated (NI) or DMSO-treated and infected samples as 100%. Three independent experiments were performed in triplicate. Data are presented as means ± SDs.

To validate the efficacy and specificity of these Wnt membrane complex inhibitors at the concentrations used to characterize infection, DKK-1, WIF-1, and Dvl-PDZ II were used to treat 293T cells transfected with TF and stimulated with Wnt3A for 20 h (gray bars) or RVFV MP12 for 5 h (blue bars) (Fig. 5A). Treatment of 293T cells with these inhibitors resulted in a dose-dependent reduction of Wnt3A-induced β-catenin Luc reporter expression. Wnt membrane complex inhibitors had no effect on RVFV MP12-induced β-catenin transcriptional activity (Fig. 5A). In contrast, Wnt inhibitors that stabilize the DC (Endo-IWR-1 and JW67) and that interfere with the interaction of β-catenin with TCF/LEF transcription factors (Wnt XII and iCRT-14) inhibited RVFV MP12-induced and Wnt3A-induced β-catenin reporter activity in 293T cells in a dose-dependent manner (Fig. 5B and C). The dose-dependent decrease in RVFV MP12-induced β-catenin reporter activity observed with these inhibitors correlates with their effect on RVFV replication, suggesting that the effect of these inhibitors on infection is specific to inhibition of Wnt/β-catenin signaling. These data indicate that the mechanism of RVFV-induced activation is downstream of the Wnt receptor complex but can be blocked by inhibitors that stabilize the DC and/or inhibitors that interfere with β-catenin-dependent transcriptional activation. Interestingly, bafilomycin A (BAF), an inhibitor of pH-dependent endocytosis, blocked RVFV MP12-induced β-catenin reporter activity, suggesting that RVFV-mediated induction occurs after virus entry (Fig. 5C).

**FIG 6:**
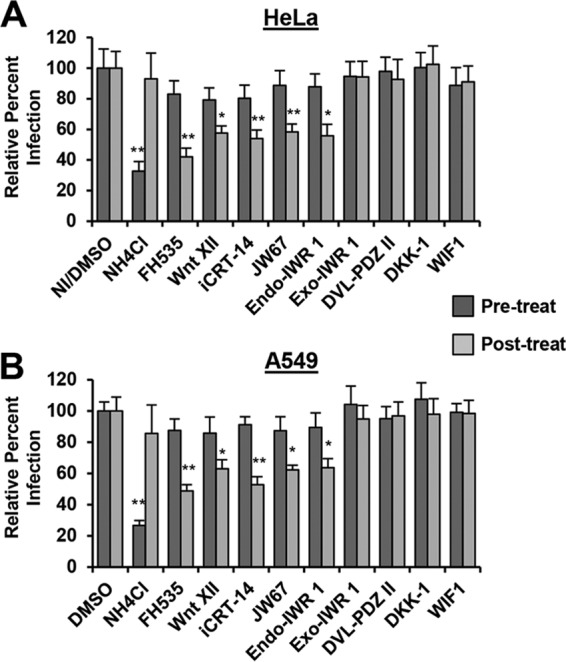
Wnt signaling inhibitors block RVFV infection at a postentry step. HeLa cells (A) or A549 cells (B) were pretreated with no inhibitor (NI)/50 μM DMSO, 50 mM NH_4_Cl, 5 μM FH535, 50 μM Dvl-PDZ II, 300 ng/ml DKK-1, 500 ng/ml WIF, or 10 μM Wnt XII, iCRT-14, JW67, Endo-IWR-1, or Exo-IWR-1 for 1 h prior to and during the 3-h infection with RVFV MP12-GFP (MOI of 1). Inhibitors were removed after the 3 h of incubation, and cells were incubated in complete medium alone overnight (pretreatment, dark gray bars). For the posttreatment condition, untreated cells were incubated with RVFV MP12-GFP (MOI of 1) for 1 h, washed with PBS to remove unbound virus, and then incubated with inhibitors in complete medium overnight (light gray bars). The percentage of infection was determined by taking NI/DMSO-treated and infected samples as 100% infected. Three independent experiments were performed in triplicate. Data are presented as means ± SDs (**, *P* < 0.01; *, *P* < 0.05).

To determine what stage of RVFV infection is dependent on Wnt/β-catenin signaling, we performed time-of-addition experiments. In our previous studies, we found that binding and entry of RVFV MP12-GFP took approximately 1 h in HeLa cells because treatment with inhibitors of endosomal acidification such as ammonium chloride (NH_4_Cl) at 1 hpi no longer blocked infection (11, 46, 47). Therefore, agents that target a postentry stage should still inhibit infection when treatments are added 1 hpi. Those inhibitors that stabilize the DC (Endo-IWR-1 and JW67) and those that interfere with activated β-catenin (FH535, Wnt XII, and iCRT-14) were shown to significantly inhibit RVFV MP12-GFP infection in HeLa cells (Fig. 6A) or A549 cells (Fig. 6B) when they were added 1 hpi and were present during the entire infection cycle. These inhibitors had little to no effect on infection when they were present 1 h prior to and during initial infection but were removed after 3 h. As expected, inhibitors that antagonize the Wnt membrane receptor complex (DKK-1, WIF-1, and Dvl-PDZ II) and the control Exo-IWR-1 had no effect on RVFV MP12-GFP infection when cells were either pretreated or treated 1 hpi. In all, these data demonstrate that inhibitors that block Wnt signaling downstream of the membrane receptor complex inhibit RVFV infection at a postentry step.

### Distantly related bunyaviruses induce Wnt/β-catenin signaling upon infection and are impeded by Wnt signaling inhibitors.

**FIG 7:**
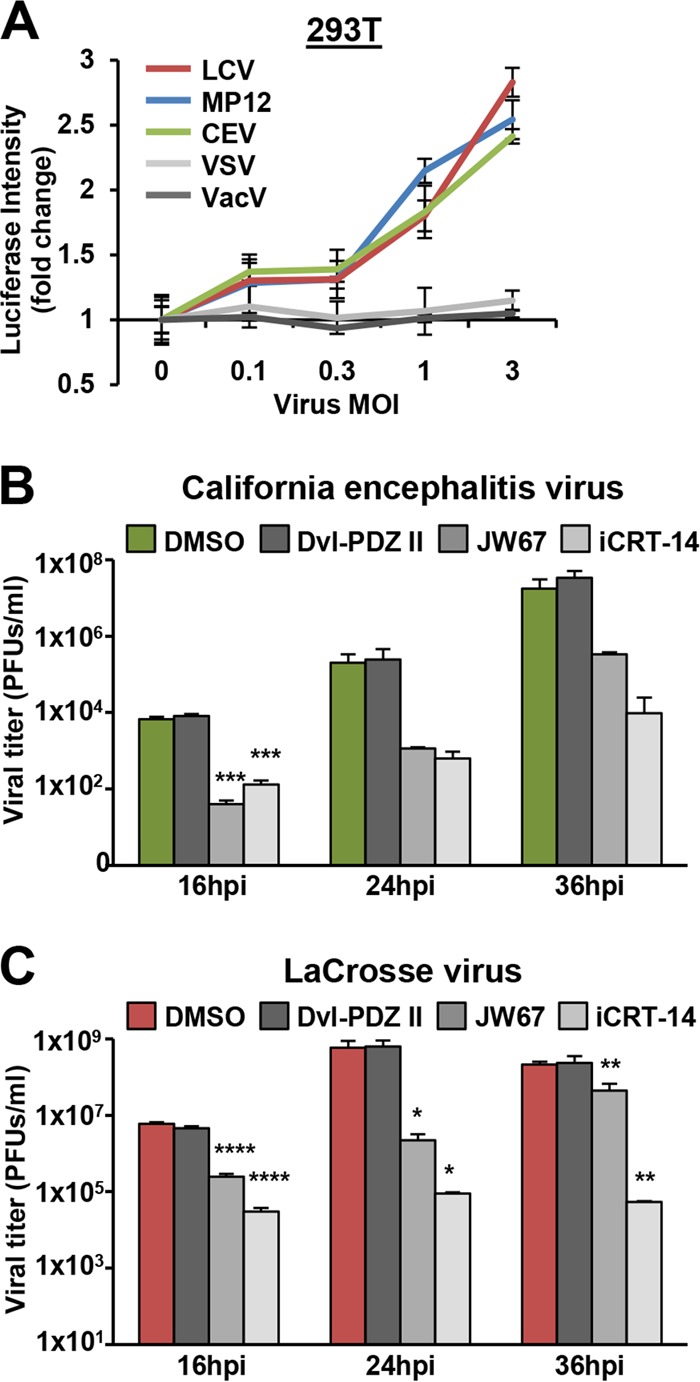
Distantly related bunyaviruses induce Wnt/β-catenin signaling upon infection and are impeded by Wnt signaling inhibitors. (A) 293T cells were transfected with TF and pcDNA3.1-GFP or pcDNA3.1-mKate reporter plasmids and then infected with viruses (RVFV MP12, LCV, CEV, VacV, or VSV) at indicated MOIs. After 5 h, luciferase activity was measured and normalized to expression of GFP (RVFV MP12, LCV, and CEV) or mKate (VacV and VSV), and fold activation was calculated by dividing the relative luciferase activity of treated/infected cells by that of untreated cells. Three independent experiments were performed in triplicate. Data are presented as means ± SDs. Representative results are shown. (B and C) HeLa cells were preincubated for 1 h with 50 μM DMSO, Dvl-PDZ II, JW67, or iCRT-14 followed by infection with CEV (B) or LCV (C) at an MOI of 0.1. The inhibitors were also present during the infection. Supernatants were collected at the indicated time points, and viral titers were measured by plaque assay on Vero cells. Data are presented as means ± SDs (*n* = 3) (****, *P* < 0.0001; ***, *P* < 0.001; **, *P* < 0.01; *, *P* < 0.05).

Although RVFV, VSV, and VacV all replicate in the cytoplasm, they do so through distinct mechanisms. Hallmark features of virus replication are typically conserved within virus families. To examine whether distantly related bunyaviruses also induce Wnt/β-catenin signaling upon infection, we performed β-catenin Luc reporter experiments in 293T cells transiently transfected with TF for 18 h and subsequently infected with California encephalitis virus (CEV) and La Crosse virus (LCV). In Fig. 7A, infections with CEV and LCV were shown to promote β-catenin reporter activation across a range of infectious doses to levels comparable to those in RVFV MP12-infected cells, while VSV and VacV infections did not induce Wnt/β-catenin signaling. Furthermore, inhibitors of Wnt/β-catenin signaling that reduced RVFV MP12-GFP, RVFV MP12, and wild-type RVFV infection (Fig. 4) also reduced CEV and LCV infections as measured by plaque assay in HeLa cells (Fig. 7B and C). More than a 50% reduction in CEV and LCV infections was exhibited when HeLa cells were treated with 50 μM JW67 or iCRT-14, whereas treatment of cells with 50 μM Dvl-PDZ II had no effect on viral titers, replicating what is seen with RVFV. These results indicate a conserved bunyaviral replication mechanism involving Wnt signaling.

## DISCUSSION

High-throughput genome-wide RNAi is a powerful tool for functional genomics with the capacity to comprehensively analyze host-pathogen interactions (48, 49). The promise of this systems approach is not only to gain a better understanding of virus-host interactions but also for the discovery of new therapeutic targets. Although this technology is not without inherent problems and pitfalls such as off-target effects and insufficient knockdown, which may lead to false positives/negative results, the analysis of the gene list as ranked functional clusters has been a successful method in gaining reproducibility between screens and providing direction for detailed cell biology investigations (50, 51). Therefore, to support the value of our final gene hit list, we validated our RNAi screening results through a thorough examination of the top-ranking cellular pathways analyzed by various bioinformatics methods, instead of the validation of individual gene hits. We used a panoply of Wnt/β-catenin signaling activation assays to demonstrate that RVFV activates the canonical Wnt signaling pathway. We demonstrated that RVFV infection activated Wnt signaling in multiple cell types optimally at 5 hpi using a β-catenin reporter assay, RVFV infection (MOI of 1 or 3) increased the level of active β-catenin protein by ∼3-fold compared to mock-infected controls (Fig. 3C), and RVFV infection increased mRNA expression of Wnt/β-catenin-responsive genes such as cyclin D1 by ∼2-fold compared to controls (Fig. 3D). Using cellular perturbation assays, we showed that RNAi-induced silencing of β-catenin expression reduced RVFV infection in multiple cell types (Fig. 2A and B) and chemical inhibition of Wnt signaling at or downstream of the DC reduced RVFV infection at a postentry step (Fig. 4 and 6). Our data demonstrating that diverse bunyaviruses induced β-catenin reporter activity and required activation of Wnt/β-catenin for productive infection suggest that activation of canonical Wnt signaling impacts replication of bunyaviruses as a family (Fig. 4 and 7).

**FIG 8:**
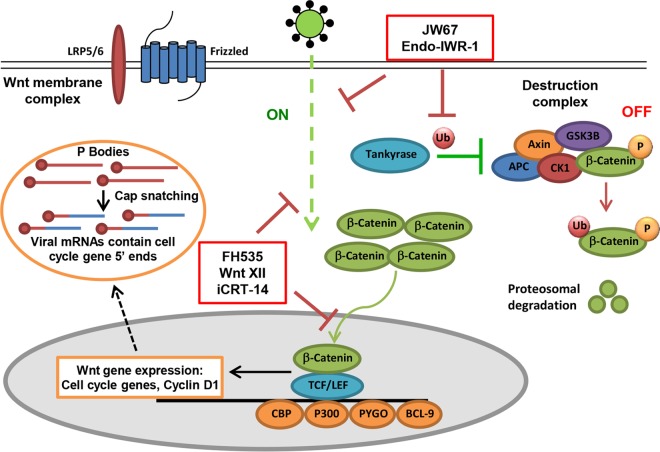
Model of bunyavirus-induced Wnt/β-catenin signaling. (Right) In the OFF state, β-catenin is sequentially phosphorylated by the destruction complex (DC) and targeted for degradation. (Left) When the DC is disrupted by regulation of the scaffolding protein axin (via recruitment to the plasma membrane by Dvl after Wnt ligand-triggered signaling or by tankyrase-mediated ADP-ribosylation of axin, resulting in its ubiquitination and degradation), the signaling pathway is in the ON state. In the ON state, β-catenin accumulates in the cytoplasm and translocates to the nucleus, where it causes activation of TCF/LEF transcription factor and subsequent transcription of β-catenin-responsive genes: cyclin D1, c-Jun, and c-Myc. Results from this study with inhibitors that target different stages of the Wnt/β-catenin signaling pathway suggest that RVFV and other bunyaviruses may induce Wnt signaling by circumventing the Wnt membrane receptor complex after virus entry, during replication (shown with dashed green line, representing proposed mechanism of virus induction of the ON state; inhibitors that decreased RVFV infection are shown in red). This leads to activation of TCF/LEF-dependent Wnt/β-catenin transcription. Bunyaviral replication is dependent on a cap-snatching mechanism where host-derived 5′ ends of mRNAs are used to prime viral gene transcription. Previous studies indicate that host-derived 5′ ends on bunyaviral mRNAs contain mostly sequences related to cell cycle genes. Wnt/β-catenin signaling may therefore be induced during viral replication in order to provide pools of mRNAs, particularly cell cycle genes, including cyclin D1, necessary for viral gene transcription initiation by cap snatching (dashed black line to represent hypothesis). Red lines in the figure represent conditions that result in the OFF state (no stimulation, stabilized DC, and inhibitor treatment), and green lines represent conditions that result in the ON state of Wnt/β-catenin signaling (tankyrase activity and virus-mediated induction). For the purposes of clarity, Wnt ligand-mediated activation of canonical Wnt signaling is not depicted in this figure.

Wnt/β-catenin signaling controls many aspects of cell behavior throughout development and in adults. One of its best-known and cancer-relevant functions is to stimulate cell proliferation. A growing body of work suggests that the cell cycle and Wnt signaling are directly linked (52). Interestingly, a recent study (12) revealed a preference for particular stages of the cell cycle for RVFV replication, as cells arrested in late S/early G_2_ phase, but not at G_1_ phase, enhanced RVFV replication. While it has not been previously reported that RVFV infection can influence shifts to preferred cell cycle stages, the Hopkins et al. study indicated that certain cell cycle stages are particularly abundant in host mRNAs available to prime bunyaviral gene transcription through a cap-snatching mechanism (12, 53). Bunyaviruses and other segmented negative-stranded RNA viruses (arenaviruses and orthomyxoviruses) cleave off the 5′ end of cellular mRNAs that includes the 5′ 7mG cap and 10 to 18 nucleotides of host mRNAs and use this fragment as a primer for viral mRNA synthesis (53). When the 5′ ends of RVFV mRNAs were sequenced, it was demonstrated that RVFV selectively snatches cell cycle-related cellular mRNAs (53). Linking these studies together with our results, it is possible that bunyaviruses activate the Wnt signaling pathway in order to induce both cell cycle shifts and abundant endogenous pools of cell cycle-related mRNAs available for priming bunyaviral gene transcription (Fig. 8). It is also interesting that cell cycle- and cyclin-related genes were among the top functional clusters identified using bioinformatics analysis of our genome-wide RNAi screening results (see Table S1 in the supplemental material). This proposed mechanism also might help explain the negative results that we found with VacV and VSV. Like many other DNA and RNA viruses that replicate in the cytoplasm, VacV and VSV both encode their own capping enzymes and therefore do not need to increase the pool of host mRNAs available for cap snatching.

It has been demonstrated that viral proteins can regulate Wnt/β-catenin signaling through direct and indirect interactions with mediators of the Wnt pathway. The HCV core protein activates Wnt/β-catenin signaling at the level of the receptor complex since a soluble FZD molecule blocks core-stimulated cell growth (31). KSHV-encoded latency-associated nuclear antigen (LANA) prevents β-catenin phosphorylation and degradation by interacting with the DC kinase GSK-3 in the nucleus and preventing its export to the cytoplasm (54). The HBV viral regulatory protein HBx suppresses GSK-3 activity via the activation of Src kinase (26). Our results with RVFV MP12-mediated activation of Wnt/β-catenin signaling, as measured by the β-catenin reporter assay and upregulation of mRNA expression of Wnt/β-catenin-responsive genes, showed that activation occurs early in infection (4 to 6 hpi) and is essentially turned off by 8 hpi (data not shown). Since the RVFV nucleoprotein (N) is the most abundant viral protein early in cell infection and is involved in gene transcription, genome replication, cap snatching, and translation (55–57), we hypothesize that it may be a determinant of Wnt/β-catenin signaling activation during infection. In addition, the amino acid sequence of N is identical between RVFV MP12, RVFV MP12-GFP, and wild-type RVFV, whereas the other viral protein transcribed early (during primary transcription) in infection, NSs, is not expressed in RVFV MP12-GFP, which was fully functional in Wnt/β-catenin signaling activation. Moreover, a recent study reported that the RVFV ZH-548 ΔNSs strain induced significant upregulation of mRNA expression of genes related to the Wnt/β-catenin signaling pathway, whereas at 8 hpi the authors did not observe the same upregulation with wild-type RVFV ZH-548 (58). Based on our results with RVFV MP12-GFP, RVFV MP12, and wild-type RVFV as well as the findings in the work of Marcato et al. (58), it is likely that NSs is not required for Wnt/β-catenin signaling activation but is responsible for repressing this signal at 8 hpi, as a result of general transcriptional shutoff (59). While we found that inhibitors downstream of the Wnt membrane receptor complex reduce RVFV infection, we cannot distinguish between direct and indirect mechanisms of Wnt activation. RVFV-mediated activation of β-catenin seems to occur downstream of receptor activation but is blocked by stabilization of the DC scaffolding protein. Besides binding to other viral proteins, RVFV N also binds to the 5′ ends of host mRNAs to initiate cap snatching, and RVFV N localizes to processing (P) bodies where cellular RNA degradation machinery is compartmentalized. Further studies should resolve whether RVFV N binding events in P bodies, where cap snatching is thought to occur, or prior virus-host interaction events in regions where Wnt signaling components are localized are the trigger for Wnt signaling activation.

Large-scale RNAi screening is a promising tool for comprehensive analysis of virus-host interactions and therapeutic target identification. To the latter point, several preclinical therapeutic agents specifically targeting the Wnt pathway have been described, and some have entered clinical trials for the treatment of a variety of cancers. PRI-724 from Prism Pharma disrupts the interaction between β-catenin and CREB-binding protein and is being studied for the treatment of solid tumors and myeloid malignancies in phase I/II clinical trials (60). Another cancer drug, called OMP-54F28 (FZD8-Fc), is an antagonist of the Wnt pathway, and results from a phase I trials were recently described (61). Preclinical studies with tankyrase inhibitors JW55 (Tocris Bioscience) and XAV939 (Novartis Pharmaceuticals) demonstrated efficacy in cellular models of cancer survival (62). In our study, the tankyrase inhibitors Endo-IWR-1 and JW67 inhibited RVFV MP12-GFP infection by greater than 50% in RVFV disease-relevant primary human hepatocytes (Fig. 4D), and JW67 significantly reduced infection of cells with wild-type RVFV, LCV, and CEV. As current and new Wnt inhibitors begin to move through safety trials, there is potential for one of these to be FDA approved, opening the door for an alternative use as an anti-RVFV therapeutic.

In all, we identified a role for canonical Wnt/β-catenin signaling during infection with diverse bunyaviruses. These studies supplement our knowledge of the fundamental mechanisms of bunyavirus gene transcription and replication and provide new avenues for countermeasure development against pathogenic bunyaviruses. We also provide the list of genome-wide RNAi screening results in order to supplement current databases of other viral RNAi screening results and to provide future direction for detailed pathway investigation from other top-ranked functional clusters not characterized in this report.

## Supplementary Material

Supplemental material

## References

[1] Elliott R, Schmaljohn CS 2013 Bunyaviridae, p 1244–1282. *In* Knipe DM, Howley PM, Cohen JI, Griffin DE, Lamb RA, Martin MA, Racaniello VR, Roizman B (ed), Fields virology, 6th ed, vol 1 Wolters Kluwer/Lippincott Williams & Wilkins, Philadelphia, PA.

[2] Pepin M, Bouloy M, Bird BH, Kemp A, Paweska J 2010 Rift Valley fever virus (Bunyaviridae: Phlebovirus): an update on pathogenesis, molecular epidemiology, vectors, diagnostics and prevention. Vet Res 41:61. doi:10.1051/vetres/2010033. 21188836 PMC2896810

[3] Rolin AI, Berrang-Ford L, Kulkarni MA 2013 The risk of Rift Valley fever virus introduction and establishment in the United States and European Union. Emerg Microbes Infect 2(12):e81. doi:10.1038/emi.2013.81. 26038446 PMC3880870

[4] Hadjihannas MV, Brückner M, Behrens J 2010 Conductin/axin2 and Wnt signalling regulates centrosome cohesion. EMBO Rep 11:317–324. doi:10.1038/embor.2010.23. 20300119 PMC2854593

[5] Ikegami T, Won S, Peters CJ, Makino S 2005 Rift Valley fever virus NSs mRNA is transcribed from an incoming anti-viral-sense S RNA segment. J Virol 79:12106–12111. doi:10.1128/JVI.79.18.12106-12111.2005. 16140788 PMC1212623

[6] Billecocq A, Spiegel M, Vialat P, Kohl A, Weber F, Bouloy M, Haller O 2004 NSs protein of Rift Valley fever virus blocks interferon production by inhibiting host gene transcription. J Virol 78:9798–9806. doi:10.1128/JVI.78.18.9798-9806.2004. 15331713 PMC514997

[7] Ikegami T, Narayanan K, Won S, Kamitani W, Peters CJ, Makino S 2009 Dual functions of Rift Valley fever virus NSs protein: inhibition of host mRNA transcription and post-transcriptional downregulation of protein kinase PKR. Ann N Y Acad Sci 1171(Suppl 1):E75–E85. doi:10.1111/j.1749-6632.2009.05054.x. 19751406 PMC3137120

[8] Ikegami T, Narayanan K, Won S, Kamitani W, Peters CJ, Makino S 2009 Rift Valley fever virus NSs protein promotes post-transcriptional downregulation of protein kinase PKR and inhibits eIF2alpha phosphorylation. PLoS Pathog 5:e1000287. doi:10.1371/journal.ppat.1000287. 19197350 PMC2629125

[9] Kalveram B, Lihoradova O, Ikegami T 2011 NSs protein of Rift Valley fever virus promotes posttranslational downregulation of the TFIIH subunit p62. J Virol 85:6234–6243. doi:10.1128/JVI.02255-10. 21543505 PMC3126510

[10] Mansuroglu Z, Josse T, Gilleron J, Billecocq A, Leger P, Bouloy M, Bonnefoy E 2010 Nonstructural NSs protein of Rift Valley fever virus interacts with pericentromeric DNA sequences of the host cell, inducing chromosome cohesion and segregation defects. J Virol 84:928–939. doi:10.1128/JVI.01165-09. 19889787 PMC2798389

[11] Harmon B, Schudel BR, Maar D, Kozina C, Ikegami T, Tseng CT, Negrete OA 2012 Rift Valley fever virus strain MP-12 enters mammalian host cells via caveola-mediated endocytosis. J Virol 86:12954–12970. doi:10.1128/JVI.02242-12. 22993156 PMC3497621

[12] Hopkins KC, McLane LM, Maqbool T, Panda D, Gordesky-Gold B, Cherry S 2013 A genome-wide RNAi screen reveals that mRNA decapping restricts bunyaviral replication by limiting the pools of Dcp2-accessible targets for cap-snatching. Genes Dev 27:1511–1525. doi:10.1101/gad.215384.113. 23824541 PMC3713431

[13] Baer A, Austin D, Narayanan A, Popova T, Kainulainen M, Bailey C, Kashanchi F, Weber F, Kehn-Hall K 2012 Induction of DNA damage signaling upon Rift Valley fever virus infection results in cell cycle arrest and increased viral replication. J Biol Chem 287:7399–7410. doi:10.1074/jbc.M111.296608. 22223653 PMC3293538

[14] Brass AL, Dykxhoorn DM, Benita Y, Yan N, Engelman A, Xavier RJ, Lieberman J, Elledge SJ 2008 Identification of host proteins required for HIV infection through a functional genomic screen. Science 319:921–926. doi:10.1126/science.1152725. 18187620

[15] Karlas A, Machuy N, Shin Y, Pleissner KP, Artarini A, Heuer D, Becker D, Khalil H, Ogilvie LA, Hess S, Maurer AP, Muller E, Wolff T, Rudel T, Meyer TF 2010 Genome-wide RNAi screen identifies human host factors crucial for influenza virus replication. Nature 463:818–822. doi:10.1038/nature08760. 20081832

[16] Krishnan MN, Ng A, Sukumaran B, Gilfoy FD, Uchil PD, Sultana H, Brass AL, Adametz R, Tsui M, Qian F, Montgomery RR, Lev S, Mason PW, Koski RA, Elledge SJ, Xavier RJ, Agaisse H, Fikrig E 2008 RNA interference screen for human genes associated with West Nile virus infection. Nature 455:242–245. doi:10.1038/nature07207. 18690214 PMC3136529

[17] Meier R, Franceschini A, Horvath P, Tetard M, Mancini R, von Mering C, Helenius A, Lozach PY 2014 Genome-wide small interfering RNA screens reveal VAMP3 as a novel host factor required for Uukuniemi virus late penetration. J Virol 88:8565–8578. doi:10.1128/JVI.00388-14. 24850728 PMC4135934

[18] MacDonald BT, Tamai K, He X 2009 Wnt/beta-catenin signaling: components, mechanisms, and diseases. Dev Cell 17:9–26. doi:10.1016/j.devcel.2009.06.016. 19619488 PMC2861485

[19] Klaus A, Birchmeier W 2008 Wnt signalling and its impact on development and cancer. Nat Rev Cancer 8:387–398. doi:10.1038/nrc2389. 18432252

[20] Voronkov A, Krauss S 2013 Wnt/beta-catenin signaling and small molecule inhibitors. Curr Pharm Des 19:634–664. doi:10.2174/138161213804581837. 23016862 PMC3529405

[21] Clevers H, Nusse R 2012 Wnt/β-catenin signaling and disease. Cell 149:1192–1205. doi:10.1016/j.cell.2012.05.012. 22682243

[22] Li VS, Ng SS, Boersema PJ, Low TY, Karthaus WR, Gerlach JP, Mohammed S, Heck AJ, Maurice MM, Mahmoudi T, Clevers H 2012 Wnt signaling through inhibition of β-catenin degradation in an intact Axin1 complex. Cell 149:1245–1256. doi:10.1016/j.cell.2012.05.002. 22682247

[23] Kahn M 2014 Can we safely target the WNT pathway? Nat Rev Drug Discov 13:513–532. doi:10.1038/nrd4233. 24981364 PMC4426976

[24] Angelova M, Zwezdaryk K, Ferris M, Shan B, Morris CA, Sullivan DE 2012 Human cytomegalovirus infection dysregulates the canonical Wnt/beta-catenin signaling pathway. PLoS Pathog 8:e1002959. doi:10.1371/journal.ppat.1002959. 23071438 PMC3469659

[25] Hsieh A, Kim HS, Lim SO, Yu DY, Jung G 2011 Hepatitis B viral X protein interacts with tumor suppressor adenomatous polyposis coli to activate Wnt/beta-catenin signaling. Cancer Lett 300:162–172. doi:10.1016/j.canlet.2010.09.018. 20971552

[26] Cha MY, Kim CM, Park YM, Ryu WS 2004 Hepatitis B virus X protein is essential for the activation of Wnt/beta-catenin signaling in hepatoma cells. Hepatology 39:1683–1693. doi:10.1002/hep.20245. 15185310

[27] Liu J, Ding X, Tang J, Cao Y, Hu P, Zhou F, Shan X, Cai X, Chen Q, Ling N, Zhang B, Bi Y, Chen K, Ren H, Huang A, He TC, Tang N 2011 Enhancement of canonical Wnt/beta-catenin signaling activity by HCV core protein promotes cell growth of hepatocellular carcinoma cells. PLoS One 6:e27496. doi:10.1371/journal.pone.0027496. 22110662 PMC3216985

[28] Al-Harthi L 2012 Interplay between Wnt/beta-catenin signaling and HIV: virologic and biologic consequences in the CNS. J Neuroimmune Pharmacol 7:731–739. doi:10.1007/s11481-012-9411-y. 23065461 PMC3518628

[29] Hayward SD, Liu J, Fujimuro M 2006 Notch and Wnt signaling: mimicry and manipulation by gamma herpesviruses. Sci STKE 2006(335):re4. 16705130 10.1126/stke.3352006re4

[30] Zhang XD, Wang Y, Ye LH 2014 Hepatitis B virus X protein accelerates the development of hepatoma. Cancer Biol Med 11:182–190. 25364579 10.7497/j.issn.2095-3941.2014.03.004 PMC4197427

[31] Liu J, Wang Z, Tang J, Tang R, Shan X, Zhang W, Chen Q, Zhou F, Chen K, Huang A, Tang N 2011 Hepatitis C virus core protein activates Wnt/beta-catenin signaling through multiple regulation of upstream molecules in the SMMC-7721 cell line. Arch Virol 156:1013–1023. doi:10.1007/s00705-011-0943-x. 21340743

[32] Kapoor A, He R, Venkatadri R, Forman M, Arav-Boger R 2013 Wnt modulating agents inhibit human cytomegalovirus replication. Antimicrob Agents Chemother 57:2761–2767. doi:10.1128/AAC.00029-13. 23571549 PMC3716142

[33] Earl PL, Americo JL, Moss B 2003 Development and use of a vaccinia virus neutralization assay based on flow cytometric detection of green fluorescent protein. J Virol 77:10684–10688. doi:10.1128/JVI.77.19.10684-10688.2003. 12970455 PMC228521

[34] Ikegami T, Won S, Peters CJ, Makino S 2006 Rescue of infectious Rift Valley fever virus entirely from cDNA, analysis of virus lacking the NSs gene, and expression of a foreign gene. J Virol 80:2933–2940. doi:10.1128/JVI.80.6.2933-2940.2006. 16501102 PMC1395455

[35] Ebert O, Shinozaki K, Huang T-G, Savontaus MJ, García-Sastre A, Woo SLC 2003 Oncolytic vesicular stomatitis virus for treatment of orthotopic hepatocellular carcinoma in immune-competent rats. Cancer Res 63:3605–3611. 12839948

[36] Zhang XD 2011 Illustration of SSMD, z score, SSMD*, z* score, and t statistic for hit selection in RNAi high-throughput screens. J Biomol Screen 16:775–785. doi:10.1177/1087057111405851. 21515799

[37] Kuri T, Habjan M, Penski N, Weber F 2010 Species-independent bioassay for sensitive quantification of antiviral type I interferons. Virol J 7:50. doi:10.1186/1743-422X-7-1. 20187932 PMC2846901

[38] Teferi WM, Dodd K, Maranchuk R, Favis N, Evans DH 2013 A whole-genome RNA interference screen for human cell factors affecting myxoma virus replication. J Virol 87:4623–4641. doi:10.1128/JVI.02617-12. 23408614 PMC3624348

[39] Nikolsky Y, Bryant J 2009 Protein networks and pathway analysis. Preface. Methods Mol Biol 563:v–vii. 19760825 10.1007/978-1-60761-175-2

[40] Huang DW, Sherman BT, Lempicki RA 2009 Systematic and integrative analysis of large gene lists using DAVID bioinformatics resources. Nat Protoc 4:44–57. doi:10.1038/nprot.2008.211. 19131956

[41] Franceschini A, Szklarczyk D, Frankild S, Kuhn M, Simonovic M, Roth A, Lin J, Minguez P, Bork P, von Mering C, Jensen LJ 2013 STRING v9.1: protein-protein interaction networks, with increased coverage and integration. Nucleic Acids Res 41:D808–D815. doi:10.1093/nar/gks1094. 23203871 PMC3531103

[42] Harmon B, Kozina C, Maar D, Carpenter TS, Branda CS, Negrete OA, Carson BD 2013 Identification of critical amino acids within the nucleoprotein of Tacaribe virus important for anti-interferon activity. J Biol Chem 288:8702–8711. doi:10.1074/jbc.M112.444760. 23382389 PMC3605688

[43] Islam MK, Baudin M, Eriksson J, Oberg C, Habjan M, Weber F, Overby AK, Ahlm C, Evander M 2016 High-throughput screening using a whole-cell virus replication reporter gene assay to identify inhibitory compounds against Rift Valley fever virus infection. J Biomol Screen 21:354–362. doi:10.1177/1087057115625184. 26762502

[44] Ross TM, Bhardwaj N, Bissel SJ, Hartman AL, Smith DR 2012 Animal models of Rift Valley fever virus infection. Virus Res 163:417–423. doi:10.1016/j.virusres.2011.10.023. 22086058

[45] Grandy D, Shan J, Zhang X, Rao S, Akunuru S, Li H, Zhang Y, Alpatov I, Zhang XA, Lang RA, Shi D-L, Zheng JJ 2009 Discovery and characterization of a small molecule inhibitor of the PDZ domain of Dishevelled. J Biol Chem 284:16256–16263. doi:10.1074/jbc.M109.009647. 19383605 PMC2713547

[46] Filone CM, Hanna SL, Caino MC, Bambina S, Doms RW, Cherry S 2010 Rift Valley fever virus infection of human cells and insect hosts is promoted by protein kinase C epsilon. PLoS One 5:e15483. doi:10.1371/journal.pone.0015483. 21124804 PMC2991366

[47] Filone CM, Heise M, Doms RW, Bertolotti-Ciarlet A 2006 Development and characterization of a Rift Valley fever virus cell-cell fusion assay using alphavirus replicon vectors. Virology 356:155–164. doi:10.1016/j.virol.2006.07.035. 16945399 PMC7134558

[48] Schudel BR, Harmon B, Abhyankar VV, Pruitt BW, Negrete OA, Singh AK 2013 Microfluidic platforms for RNA interference screening of virus-host interactions. Lab Chip 13:811–817. doi:10.1039/c2lc41165b. 23361404

[49] Cherry S 2009 What have RNAi screens taught us about viral-host interactions? Curr Opin Microbiol 12:446–452. doi:10.1016/j.mib.2009.06.002. 19576842 PMC2766546

[50] Bushman FD, Malani N, Fernandes J, D'Orso I, Cagney G, Diamond TL, Zhou H, Hazuda DJ, Espeseth AS, Konig R, Bandyopadhyay S, Ideker T, Goff SP, Krogan NJ, Frankel AD, Young JA, Chanda SK 2009 Host cell factors in HIV replication: meta-analysis of genome-wide studies. PLoS Pathog 5:e1000437. doi:10.1371/journal.ppat.1000437. 19478882 PMC2682202

[51] Mercer J, Snijder B, Sacher R, Burkard C, Bleck CK, Stahlberg H, Pelkmans L, Helenius A 2012 RNAi screening reveals proteasome- and Cullin3-dependent stages in vaccinia virus infection. Cell Rep 2:1036–1047. doi:10.1016/j.celrep.2012.09.003. 23084750

[52] Davidson G, Niehrs C 2010 Emerging links between CDK cell cycle regulators and Wnt signaling. Trends Cell Biol 20:453–460. doi:10.1016/j.tcb.2010.05.002. 20627573

[53] Hopkins K, Cherry S 2013 Bunyaviral cap-snatching vs. decapping: recycling cell cycle mRNAs. Cell Cycle 12:3711–3712. doi:10.4161/cc.26878. 24145225 PMC3905058

[54] Fujimuro M, Liu J, Zhu J, Yokosawa H, Hayward SD 2005 Regulation of the interaction between glycogen synthase kinase 3 and the Kaposi's sarcoma-associated herpesvirus latency-associated nuclear antigen. J Virol 79:10429–10441. doi:10.1128/JVI.79.16.10429-10441.2005. 16051835 PMC1182668

[55] Le May N, Gauliard N, Billecocq A, Bouloy M 2005 The N terminus of Rift Valley fever virus nucleoprotein is essential for dimerization. J Virol 79:11974–11980. doi:10.1128/JVI.79.18.11974-11980.2005. 16140773 PMC1212621

[56] Cheng E, Mir MA 2012 Signatures of host mRNA 5′ terminus for efficient hantavirus cap snatching. J Virol 86:10173–10185. doi:10.1128/JVI.05560-11. 22787213 PMC3446632

[57] Guu TS, Zheng W, Tao YJ 2012 Bunyavirus: structure and replication. Adv Exp Med Biol 726:245–266. doi:10.1007/978-1-4614-0980-9_11. 22297517

[58] Marcato V, Luron L, Laqueuvre LM, Simon D, Mansuroglu Z, Flamand M, Panthier JJ, Souès S, Massaad C, Bonnefoy E 2016 β-Catenin upregulates the constitutive and virus-induced transcriptional capacity of the interferon beta promoter through T-cell factor binding sites. Mol Cell Biol 36:13–29. doi:10.1128/MCB.00641-15. 26459757 PMC4702592

[59] Ikegami T, Makino S 2011 The pathogenesis of Rift Valley fever. Viruses 3:493–519. doi:10.3390/v3050493. 21666766 PMC3111045

[60] Lenz HJ, Kahn M 2014 Safely targeting cancer stem cells via selective catenin coactivator antagonism. Cancer Sci 105:1087–1092. doi:10.1111/cas.12471. 24975284 PMC4175086

[61] Le PN, McDermott JD, Jimeno A 2015 Targeting the Wnt pathway in human cancers: therapeutic targeting with a focus on OMP-54F28. Pharmacol Ther 146:1–11. doi:10.1016/j.pharmthera.2014.08.005. 25172549 PMC4304994

[62] Waaler J, Machon O, Tumova L, Dinh H, Korinek V, Wilson SR, Paulsen JE, Pedersen NM, Eide TJ, Machonova O, Gradl D, Voronkov A, von Kries JP, Krauss S 2012 A novel tankyrase inhibitor decreases canonical Wnt signaling in colon carcinoma cells and reduces tumor growth in conditional APC mutant mice. Cancer Res 72:2822–2832. doi:10.1158/0008-5472.CAN-11-3336. 22440753

