# Supplementary material for: A Genome-Wide RNA Interference Screen Identifies a Role for Wnt/β-Catenin Signaling during Rift Valley Fever Virus Infection
Source: J Virol. 2016 Jul 27;90(16):7084–97. doi: 10.1128/JVI.00543-16 (PMC4984662; doi:10.1128/JVI.00543-16)
Supplement: Supplemental material [file supp_90_16_7084__index.html]

A Genome-Wide RNA Interference Screen Identifies a Role for Wnt/β-Catenin Signaling during Rift Valley Fever Virus Infection — Supplemental material 

# A Genome-Wide RNA Interference Screen Identifies a Role for Wnt/β-Catenin Signaling during Rift Valley Fever Virus Infection

## Supplemental material

- Supplemental file 1 -

  Table S1 (RNAi screening data set and pathway analysis.)

  XLSX, 84K
